# Measurement variability of right atrial and ventricular monophasic action potential and refractory period measurements in the standing non-sedated horse

**DOI:** 10.1186/s12917-018-1399-y

**Published:** 2018-03-20

**Authors:** Dominique De Clercq, Barbara Broux, Lisse Vera, Annelies Decloedt, Gunther van Loon

**Affiliations:** Department of Large Animal Internal Medicine, Ghent University, Salisburylaan 133, B-9820 Merelbeke, Belgium

**Keywords:** Electrophysiology, Echocardiography, Monophasic action potential, Equine

## Abstract

**Background:**

In human and veterinary medicine, monophasic action potential (MAP) analysis and determination of local refractory periods by contact electrode technique gives valuable information about local cardiac electrophysiological properties. It is used to investigate dysrhythmias and the impact of drugs on the myocardium. Precise measurement of total MAP duration is difficult, therefore the MAP duration is usually determined at a repolarization level of 90% (APD90). Until now, no studies are published about the feasibility of this technique in the standing non-sedated horse. In 6 healthy Warmblood horses, on two different days, an 8F quadripolar contact catheter was passed through a jugular introducer sheath and placed under ultrasound guidance at the level of the intervenous tubercle or right atrial free wall (RA), and in the right ventricular apex (RV) to record the MAP. The MAP amplitude and APD90 were measured at a resting sinus rhythm (heart rate of 30-42 bpm) and at pacing cycle lengths (PCL) of 1000 and 600 ms. The effective refractory period (ERP) was determined at PCL of 1000 and 600 ms.

**Results:**

The overall mean (±SD) APD90 (rest), APD90 (1000) and APD90 (600) were 263 ± 39 ms, 262 ± 41 ms, 236 ± 47 ms for the RA and 467 ± 23 ms, 412 ± 38 ms, 322 ± 29 ms for the RV. The mean ERP1000 and ERP600 were 273 ± 24 ms and 256 ± 22 ms for the RA and 386 ± 40 ms and 293 ± 30 ms for the RV. The measurement variability for the amplitude, APD90 and ERP measurements in the RA ranged between 36 and 44, 9–22 and 7–8%, respectively. The measurement variability for the amplitude, APD90 and ERP measurements in the RV ranged between 49 and 66, 6–7 and 10–12%, respectively.

**Conclusions:**

RA and RV MAP duration and ERP can be obtained by a contact electrode in standing non-sedated horses. The measurement variability varies with catheter location.

## Background

Electrocardiography (ECG) is the most commonly used technique for recording electrical activity of the myocardium. However, it does not provide precise information regarding the dispersion of repolarization or after-depolarizations since an ECG represents the summation of electrical activity of many myocardial cells from a relatively large region of the heart [1]. Local activity can be recorded by an intracellular action potential (IAP). However, IAP recordings can only be made in an individual cardiac cell and thus can only be performed in vitro [2]. Monophasic action potential (MAP) has a smaller magnitude than the IAP but it is proven that MAP recordings can give highly accurate information about local atrial or ventricular activation time and the entire time course of myocardial repolarization [3–11]. The local electrical activity of the in situ beating heart can be assessed by monophasic action potentials. This waveform can be obtained by slight pressure of a contact electrode on the heart muscle and is a result of membrane potential discharges (depolarisation) and slowly recharges (repolarisation). MAPs are useful for investigation of the electrophysiology of different dysrhythmias (i.e. effects of cycle length changes and the role of afterdepolarizations in the genesis of triggered dysrhythmias) and the impact of drugs (i.e. the effect of antiarrhythmic drugs on the action potential duration in the in situ heart) on the heart of different species [3–11]. The recording of MAP signals in awake dogs without any interference of anaesthetic drugs is possible. With this procedure the effect of anaesthetics drugs on the autonomic nervous system, which has an important impact on cardiac electrophysiology, can be avoided [12, 13]. The underlying principles concerning MAP recordings are described elsewhere [1, 14, 15].

Until now, very little information about the possibility of making MAP recordings in standing horses is available. This paper describes the technique and the day-to-day variability of right cardiac MAP recording and refractory period measurement in the standing non-sedated horse.

## Methods

The experimental protocol was approved by the Ethical Committee of the Faculty of Veterinary Medicine and the Faculty of Bioscience Engineering at Ghent University (case number EC 2015/85, date of approval 9th of September, 2015). The horses used in this study are Faculty owned horses.

**Fig. 1:**
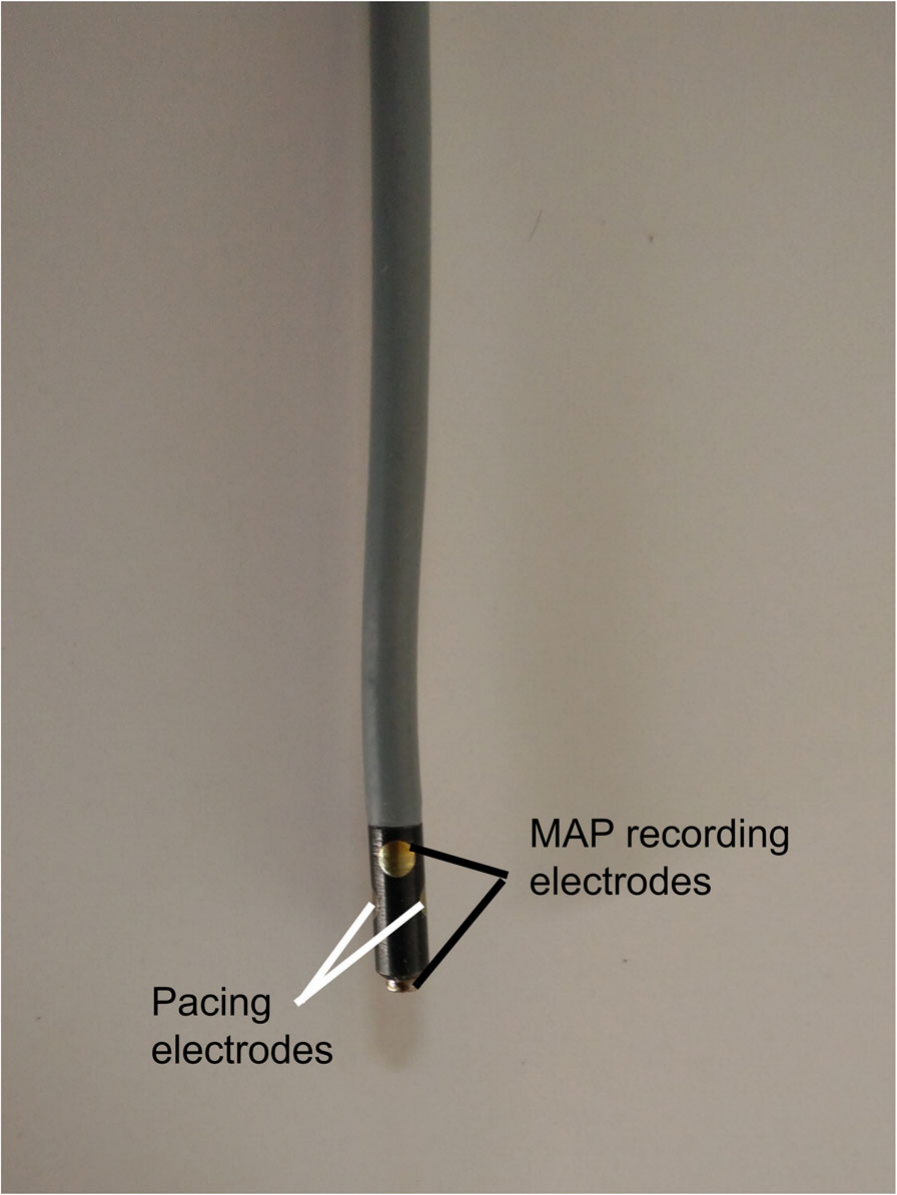
Illustration of the tip of an 8F quadripolar contact catheter with separated recording and pacing electrodes

**Fig. 2:**
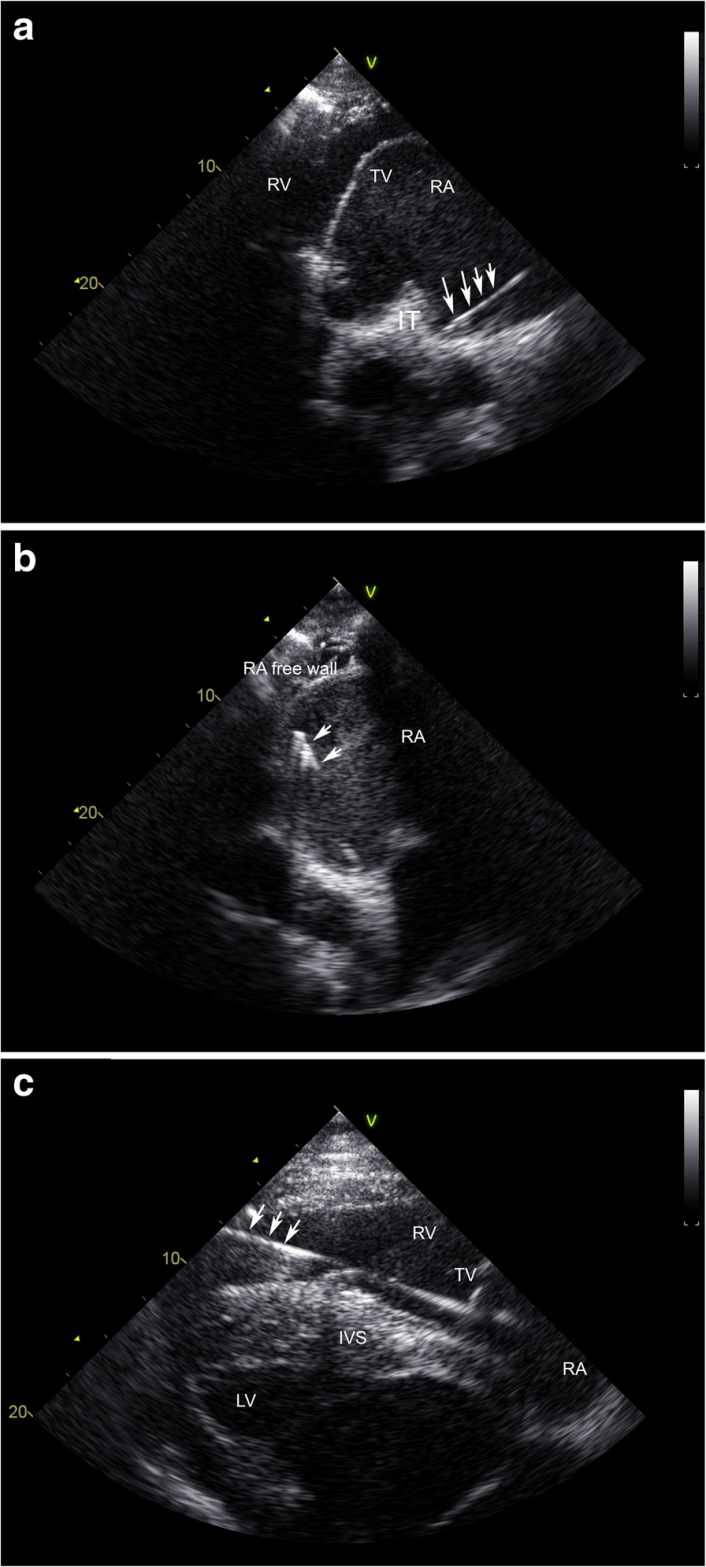
Ultrasound images of the contact MAP electrode (arrow) positioned at the level of the intervenous tubercle (IT) in the right atrium (**a**), at the level of the right atrial free wall (**b**) and in the right ventricular apex (**c**). RA: right atrium, TV: tricuspid valve, RV: right ventricle, LV: left ventricle, IVS: interventricular septum

The study population consisted of 6 healthy warmblood horses (3 geldings, 3 mares) aged (mean ± standard deviation) 10 ± 5 years, with a body weight of 531 ± 70 kg and a height at the withers of 160 ± 6 cm. Cardiovascular or other diseases were excluded based on a physical examination, a complete blood exam, an echocardiographic examination, a 30-min resting ECG and a 20-min standardized exercise ECG. All horses underwent the procedure two times on two different days with at least 2 weeks interval. In each horse an 8.5F introducer sheath (Introflex, Edwards Lifesciences, Dilbeek, Belgium) was placed using the Seldinger technique in the lower third of the jugular vein. An 8F quadripolar contact catheter with separated recording and pacing electrodes (EasyMap MAP, Medfact, Lörrach, Germany) was used [3, 5] (Fig. 1). The catheter was inserted via the introducer sheath and placed under ultrasound guidance at the level of the intervenous tubercle (in 9/12 procedures) or at the level of the right atrial free wall (RA) (in 3/12 procedures) and in the right ventricular apex (RV) (in all procedures =12/12) (Fig. 2). The catheter tip was pressed softly against the endocardium. Exact pressure determination against the wall was not possible with this type of catheter. MAPs were recorded after placement of the catheter in a position that provided continuous recordings with a stable amplitude of > 2 mV in the RA and > 3 mV in the RV, a stable baseline, a sharp positive phase and an upward convex plateau phase [14]. MAP signal and a surface ECG (base-apex) were recorded simultaneously using a multichannel recorder (PowerLab 8/35, ADInstruments, Oxford, United Kingdom) with amplifier (Bio Amp and Quad Bridge Amp, ADInstruments, Oxford, United Kingdom) and digitized on a computer. Measurements were performed off-line using the ECG analysis (used for determination of QT interval) and cardiac action potential peak analysis module of a specialized semi-automatic data analysis software program (LabChart 8, ADInstruments, Oxford, United Kingdom). The minimum peak height detection was set at 1.5 mV and an automatic resting membrane potential detection was used. The MAP signals were amplified using a low-pass filter with settings between 20 and 50 Hz because it reduces noise which results in a smoother signal. Each MAP curve was checked separately and only MAPs where the automatic resting membrane potential was automatically set at the level of true baseline were used. In every horse, 15 cardiac cycles were analysed per chamber and per day. The APD90 was automatically measured by the software as the duration between the upstroke and 90% repolarization. The amplitude was measured as the height from baseline to the crest of the plateau phase. First, measurements were made in sinus rhythm at rest (non-paced) at a cardiac rate between 30 and 42 beats per minute (bpm). Subsequently, to have a more stable rhythm and to follow the impact of heart frequency on action potential characteristics, the pacing electrodes of the MAP catheter were connected to a pacemaker programmer (Medtronic, Jette, Belgium) and pacing was performed at a pacing cycle length of 1000 and 600 ms to record MAPs at each pacing rate. Finally, atrial and ventricular effective refractory period (ERP) were determined as previously described [16, 17]. In brief, after pacing for 2 min with a fixed pacing interval (S1-S1), an extra stimulus (S2), at two times threshold amplitude, was introduced with a coupling interval (S1-S2) below the expected refractory period. The coupling interval was increased in steps of 8 ms until capture of the extra stimulus occurred (the atrial or ventricular S2 was followed by a P wave or QRS complex on the surface ECG, respectively). The longest S1-S2 interval without capture was taken as the effective refractory period. ERP was always determined 5 times to obtain a mean value. After the procedure, all catheters were gently removed under ultrasound guidance.

### Data analysis

Statistical analyses were performed using dedicated software (SPSS Statistics 22.0, SPSS Inc., Chicago, Illinois). Mean and standard deviation (SD) were calculated from pooled measurements of all examinations for each horse. Measurement variability was obtained by comparing the results of the measurements from day 1 and day 2 in a one-way repeated measures analysis of variance with the horse as the unit of repeated measure. The numerical values for the reported coefficients of variation (CV) were calculated by dividing the square root of the mean square error (MSE) by the grand mean, multiplied by 100. The relationship between QT interval and APD90 was evaluated using a linear regression model with QT interval, day and horse as predictors. A value of *P* < 0.05 was considered significant.

## Results

**Fig. 3:**
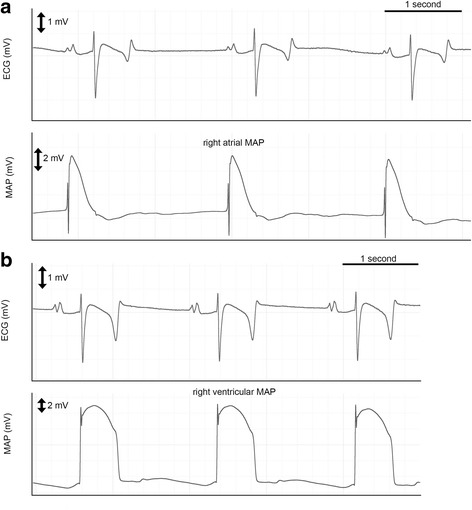
Surface electrocardiogram (ECG) with corresponding right atrial (**a**) and right ventricular (**b**) monophasic action potential (MAP) at resting sinus heart rhythm in the standing non-sedated horse

**Fig. 4:**
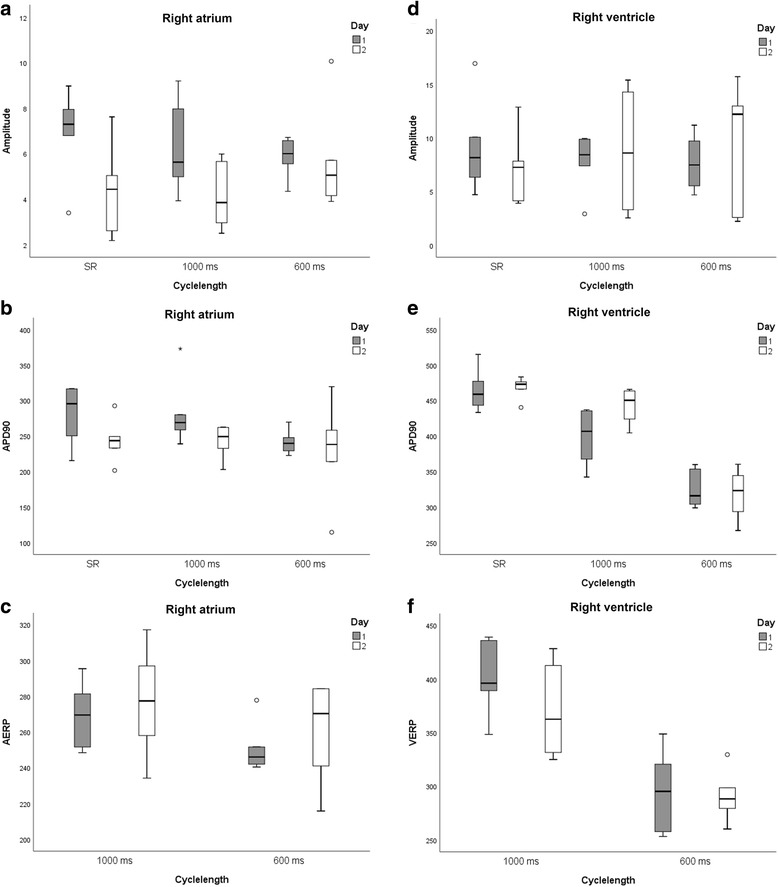
Graphical illustration of monophasic action potential amplitude (**a** and **d**) and repolarization level of 90% (APD90) (**b** and **e**), and effective refractory periods (ERP) (**c** and **f**) measured at rest (sinus rhythm: SR) and at a pacing cycle length of 1000 and 600 ms in the right atrium (RA) (**a**, **b** and **c**) and right ventricle (RV) (**d**, **e** and **f**) in two different days. The median and spread of the value is indicated by a boxplot, with the box span indicating the middle half of the observations, the line in the box marking the median, and the whiskers indicating the range of observations. Extreme values were plotted individually at the end of the whiskers

Right atrial (Fig. 3a) and right ventricular (Fig. 3b) MAP recordings of sufficient quality could be obtained and analysed in non-sedated standing horses. However, horse movements, occasionally resulted in loss of contact requiring MAP catheter repositioning. The total recording time per horse and per day ranged between 35 and 165 min. Catheter positioning was occasionally associated with self-limiting atrial or ventricular depolarizations which terminated after slight catheter tip movements. During atrial ERP measurements, a short episodes of self-limiting episode of atrial tachycardia was occasionally found and 1 horse showed a 20-min paroxysm of atrial fibrillation. Ventricular ERP measurements were never associated with tachyarrhythmias. No complications were observed after the procedure. Typical right atrial and ventricular MAP recordings are shown in Fig. 3. All the results of the two different days are shown in Table 1 and Fig. 4. In 1 horse a difference of up to 100 ms was observed during VERP1000 measurement. The QT interval was a significant predictor of APD90 in sinus rhythm (beta 0.604, *P* < 0.001) and at a pacing cycle length of 1000 ms (beta − 0.177, *P* = 0.018), but not at a pacing cycle length of 600 ms.

**Table 1:** Mean ± SD of monophasic action potential amplitude and repolarization level of 90% (APD90), and effective refractory periods (ERP) measured at rest and at a pacing cycle length of 1000 and 600 ms in the right atrium and right ventricle

	Right atrium				Right ventricle			
	Mean ± SD				Mean ± SD			
	Day 1	Day 2	Overall	Cv %	Day 1	Day 2	Overall	CV %
amplitude (rest) (mV)	6.9 ± 1.9	4.4 ± 1.9	5.7 ± 2.3	35.6	9.1 ± 4.3	7.2 ± 3.3	8.1 ± 3.8	49.2
amplitude (1000) (mV)	6.0 ± 2.0	4.0 ± 1.0	5.0 ± 2.0	44.0	7.8 ± 2.6	8.8 ± 5.4	8.3 ± 4.0	58.9
amplitude (600) (mV)	6.0 ± 1.0	6.0 ± 2.0	6.0 ± 2.0	37.3	7.7 ± 2.5	9.7 ± .8	8.7 ± 4.4	65.5
APD90 (rest) (ms)	282 ± 41	244 ± 29	263 ± 39	9.1	464 ± 30	469 ± 15	467 ± 23	6.2
APD90 (1000) (ms)	281 ± 47	243 ± 23	262 ± 41	12.3	399 ± 38	443 ± 24	421 ± 38	7.3
APD90 (600) (ms)	242 ± 17	231 ± 67	236 ± 47	21.6	325 ± 26	319 ± 34	322 ± 29	5.6
ERP1000 (ms)	269 ± 18	277 ± 31	273 ± 24	7.9	401 ± 34	370 ± 43	386 ± 40	11.8
ERP600 (ms)	251 ± 14	261 ± 28	256 ± 22	6.5	295 ± 38	291 ± 23	293 ± 30	9.6

## Discussion

The present paper describes the technique to determine right atrial and right ventricular repolarization and refractoriness in the standing non-sedated horse. In humans, dogs, cats and pigs, MAP recordings play an important role in the investigation of myocardial electrophysiology under physiological, pathological and pharmacological conditions and have a low level of risk [18–21]. However, complications such as sustained dysrhythmias, movement artefacts or pericardial effusion have been reported [21]. In our study, complications were limited to some short episodes of ectopic rhythms and 1 short episode of atrial fibrillation during ERP measurement. The ERP values in our study were comparable with previously reported results [17]. The coefficient of variation of the ERP measurements ranged between 7 and 12% but in one horse the difference in VERP1000 measurement was > 100 ms. As observed in awake dogs, neck and body movements of the horse occasionally resulted in dislocation of the catheter and loss of tip pressure increasing recording time and measurement variability [12]. Due to a more fixed catheter position in the apex, dislodgement of the catheter tip was less frequently observed in ventricular MAP recordings. Probably, some spatial repolarization differences might also contribute to the measurement variability [12, 18]. MAP duration and amplitude can be variable due to angle differences of the catheter tip relative to the myocardium, the catheter location and varying endocardial contact pressures in the beating heart [14, 22]. Prolonged good quality MAP recordings from the same endocardial site (up to 1 h), as described for awake dogs, could not be achieved in our horses [13]. Mild sedation or the use of another type of catheter could have facilitated the procedure in our study. In humans and sedated dogs, the MAP amplitude of the atrium and ventricle ranges between 5 and 50 mV due to a variability in contact pressure but also due to tissue type and species [12, 14, 22]. The difference in amplitude between the atrium and the ventricle has partly been explained by the different thickness of the endocardial tissue beneath the tip of the catheter [12]. The reason for the species- and tissue-related difference in MAP amplitude remains unexplained [21]. Intracellular recordings have amplitudes of approximately 120 mV regardless species or tissue dependence [22]. The MAP amplitude in our horses ranged between 2 and 10 mV for the atrium and between 3 and 16 mV for the ventricle, which could be related to endocardial properties. The large size of the equine heart seems to be a large disadvantage and also has probably contributed to a less stable catheter position. The most frequently used filters with MAP measurements are the low-pass filter and the high-pass filter. The manuals of the used program advice to use a low-pass filter since it lets low frequencies pass and stops high frequencies. This results in a reduction of noise, it gives a smoother MAP signal. The use of a high pass filter lets high frequencies pass and remove any steady component or slow fluctuations from the signal. This setting is used to stabilize baseline. In this study, a low pass filter was used to obtain smoother MAP signals. In addition, each MAP signal was checked manually to evaluate if the automatic resting membrane potential was correctly set at the level of baseline.

### Study limitations

Despite ultrasonographic guidance, dislocation of the catheter and changes in contact pressure by the beating heart and horse movements contributed to the variability. Further research is necessary to identify whether repeating the measurements in a higher number of horses, harder pressure on the catheter tip, measurement under general anaesthesia or using another type of catheter (with i.e. a spring-steel stylet that is inserted into the myocardium) results in a lower variability in horses. Also the influence of spatial differences within right atrium and right ventricle, and between the left and right heart should be investigated. In humans, access to the left heart is possible transseptal or via the arteria femoralis [23–25]. In horses, access to the left heart might be possible via the carotid artery or transseptal.

The present study describes the feasibility and measurement variability of MAP recordings and refractory period measurements with a quadripolar contact catheter in the standing non-sedated horse. Further research is necessary to evaluate if this MAP recording technique might be useful to investigate equine dysrhythmias and the impact of drugs on the myocardium.

## Conclusion

RA and RV MAP duration and ERP can be obtained by a contact electrode in standing non-sedated horses. The measurement variability varies with catheter location.

## Abbreviations

APD90: Action potential at a repolarization of 90%
bpm: Beats per minute
CV: Coefficient of variation
ECG: Electrocardiography
ERP: Effective refractory period
Hz: Hertz
MAP: Monophasic action potential
ms: Milliseconds
MSE: Mean square error
mV: Millivolt
PLC: Pacing cycle length
RA: Right atrium
RV: Right ventricle
SD: Standard deviation
